# INFLUENCE OF PATIENT AGE AND COLORECTAL POLYP SIZE ON HISTOPATHOLOGY FINDINGS

**DOI:** 10.1590/S0102-67202014000200006

**Published:** 2014

**Authors:** Silvana Marques e SILVA, Viviane Fernandes ROSA, Antônio Carlos Nóbrega dos SANTOS, Romulo Medeiros de ALMEIDA, Paulo Gonçalves de OLIVEIRA, João Batista de SOUSA

**Keywords:** Colonoscopy, Polyps, Adenoma, Colon, Colorectal neoplasms

## Abstract

**Background:**

Colorectal cancer is a major cause of morbidity and mortality and can arise through the adenoma-carcinoma sequence. Colonoscopy is considered the method of choice for population-wide cancer screening.

**Aim:**

To assess the characteristics of endoscopically resected polyps in a consecutive series of patients who underwent colonoscopy at a university hospital and compare histopathology findings according to patient age and polyp size.

**Methods:**

Retrospective, cross-sectional of 1950 colonoscopy reports from consecutively examined patients. The sample was restricted to reports that mentioned colorectal polyps. A chart review was carried out for collection of demographic data and histopathology results. Data were compared for polyps sized ≤0.5 cm and ≥0.6 cm and then for polyps sized ≤1.0 cm and ≥1.1 cm. Finally, all polyps resected from patients aged 49 years or younger were compared with those resected from patients aged 50 years or older.

**Results:**

A total of 272 colorectal polyps were resected in 224 of the 1950 colonoscopies included in the sample (11.5%). Polyps >1 cm tended to be pedunculated (p=0.000) and were more likely to exhibit an adenomatous component (p=0.001), a villous component (p=0.000), and dysplasia (p=0.003). These findings held true when the size cutoff was set at 0.5 cm. Patients aged 50 years or older were more likely to have sessile polyps (p=0.023) and polyps located in the proximal colon (p=0.009). There were no significant differences between groups in histopathology or presence of dysplasia.

**Conclusion:**

Polyp size is associated with presence of adenomas, a villous component, and dysplasia, whereas patient age is more frequently associated with sessile polyps in the proximal colon.

## Abstract (pt)

**Racional:**

O câncer colorretal é causa importante de morbimortalidade e pode desenvolver-se pela sequência adenoma-carcinoma. A videocolonoscopia é considerada método de escolha para rastreamento populacional para esta neoplasia.

**Objetivo:**

Avaliar as características de pólipos endoscopicamente ressecados em uma série consecutiva de pacientes submetidos à videocolonoscopia em um hospital universitário e comparar os achados histopatológicos de acordo com a idade do paciente e o tamanho dos pólipos.

**Método:**

Estudo retrospectivo transversal baseado na análise dos laudos de 1950 videocolonoscopias realizadas consecutivamente. Foram selecionados aqueles em que foram evidenciados pólipos no cólon ou reto. Procedeu-se a revisão dos prontuários para coleta de dados demográficos e da avaliação histopatológica dos espécimes. Foram comparados os achados relativos aos pólipos de até 0,5 cm com os acima de 0,6 cm. Posteriormente, foram comparados pólipos de até 1 cm com os acima de 1,1 cm. Em um terceiro momento foram realizadas comparações dos achados dos pólipos ressecados de pacientes com idade até 49 anos com aqueles retirados de pacientes acima de 50 anos.

**Resultados:**

Foram ressecados pólipos colorretais em 224 dos 1950 exames avaliados (11,5%), com retirada total de 272 pólipos. Pólipos maiores de 1 cm tenderam a ser pediculados (p=0,000) e tiveram maior chance de apresentarem componente adenomatoso (p=0,001), componente viloso (p=0,000) e displasia (p=0,003). Os mesmos achados foram observados com ponto de corte de 0,5 cm. Pacientes com 50 anos ou mais apresentaram mais frequentemente pólipos sésseis (p=0,023) e localizados no cólon proximal (p=0,009). Não houve diferença significante entre os grupos em relação à histopatologia ou presença de displasia.

**Conclusão:**

O tamanho dos pólipos está mais associado à ocorrência de adenomas, presença de componente viloso e de displasia. Já a idade relaciona-se mais frequentemente com a ocorrência de pólipos sésseis e de localização proximal.

## INTRODUCTION

Colorectal cancer (CRC) is a major cause of morbidity and mortality. It is the fourth most common malignant neoplasm and the third leading cause of cancer mortality in Brazil^1^. The incidence is higher between the ages of 50 and 70.

It is widely known that 60-90% of this cancer arise from adenomas^8^, through the adenoma-carcinoma sequence. In the majority of cases, this transformation is relatively slow, taking up to 10-15 years^8^. This slow growth enables prevention of CRC by endoscopic resection of polyps.

In view of its prevalence, its long asymptomatic interval, and the presence of treatable precancerous lesions, CRC fulfills all criteria for routine population-wide screening. Colonoscopy is considered the method of choice for this purpose^20^. Randomized clinical trials and several cohort studies have shown that colonoscopic polypectomy reduces its incidence by 76-90%, as compared with a general population registry^22,29^.

Colorectal adenomas are the neoplasms most commonly detected during screening colonoscopy, as well as in diagnostic colonoscopy of symptomatic patients over the age of 50^28^. Adenomatous polyps may be classified as low-, moderate-, or high-risk lesions in terms of the risk of progression to cancer^29^. Lesions are considered advanced when they are ≥1 cm in size or exhibit a villous component or high-grade dysplasia^26^. Age is considered a risk factor for the presence of adenomas and dysplasia, the incidence of which increases once the sixth decade of life is reached^23^.

The objective of this study was to assess the characteristics of polyps resected endoscopically from a consecutive series of patients who underwent colonoscopy at a university hospital and compare histopathological findings by patient age and polyp size.

## METHODS

This was a retrospective, cross-sectional, chart review study based on analysis of the reports of 1950 colonoscopies performed consecutively at the Coloproctology Service of Hospital Universitário de Brasília. The indications for colonoscopy were not taken into account. Reports were obtained from the hospital database. Only those that described evidence of polyps in the colon or rectum were considered for analysis. Patient charts were then reviewed to collect demographic data and the results of histopathological examination of resected specimens. Each polyp was analyzed individually, even when several were resected from the same patient.

Patients with inflammatory bowel disease, colorectal malignancy, or genetic syndromes associated with polyposis were excluded from the sample, as were incomplete colonoscopies, polyps with malignant transformation, and unresected polyps.

Initially, the histopathological features of resected polyps were compared according to polyp size, defined as a dichotomous variable (≤0.5 cm or ≥0.6 cm as estimated by the endoscopist). A second analysis then compared ≤1.0 cm and ≥1.1 cm polyps. Finally, all polyps, regardless of size, resected from patients aged 49 years or younger were compared with those resected from patients aged 50 years or older.

Polyps located proximal to the splenic flexure of the colon were considered proximal, whereas those located after the splenic flexure were distal.

Statistical analyses were performed in the SPSS 17.0 software environment. Fisher's exact test was used for between-group comparisons. The significance level was set at p<0.05.

## RESULTS

A total of 272 colorectal polyps were resected in 224 of the 1950 colonoscopies included in the sample (11.5%). Most of these colonoscopies had been performed in women (55.1%), and 75.9% of patients were aged 50 years or older.

Polyps were solitary in 51% of cases. In terms of morphology, 79.8% were sessile and 20.2 % were pedunculated (stalked). The most frequent site was the left colon (43.4%), followed by the right colon (20.6%), the transverse colon (17.6%), and the rectum (17.6%). Polyps were scattered throughout the colon in 7% of cases. Most polyps were <1 cm in size according to the examining physician (88.6%). The polyp size estimated by the endoscopist matched that determined by the pathologist in 80.1% of cases.

On histopathological examination, 42.6% of polyps were tubular adenomas, 2.9% were villous adenomas, 7% were tubulovillous adenomas, 23.2% were hyperplastic, 13% were inflammatory, and 4% were hamartomas. Other diagnoses were established in 7.3% of cases.

Comparison of polyps by size, using 1 cm as a cutoff, showed that larger polyps tended to be pedunculated (p=0.000) and were more likely to exhibit an adenomatous component (p=0.001) and dysplasia (p=0.003). There were no between-group differences in the distribution of polyp sites (p=0.677, Table 1).

**TABLE 1:** Characteristics of resected polyps according to size (≤ 1 cm v ersus ≥1.1 cm)

Polyp characteristics			Polyp size				p
			0 to 1 cm		≥1.1 cm		
			N	%	N	%	
Morphology	Sessile		207	85.9	10	32.3	0.000
	Pedunculated		34	14.1	21	67.7	
Site	Distal		148	61.4	20	64.5	0.845
	Proximal		93	38.6	11	35.5	
Histopathology	Adenoma		118	48.9	25	80.6	0.001
	Other		123	51.1	6	19.4	
Dysplasia	Present		115	47.7	25	80.6	0.003
	Grade	Low	105	43.6	17	54.7	
		Moderate	10	4.1	7	22.6	
		High	0	0	1	3.3	
	Absent		120	49.8	6	19.4	
	Indeterminate		6	2.5	0	0	

Histopathology findings according to polyp size are described in Table 2. Only 10.7% of adenomas ≤1 cm in size had a villous component, versus 56% of those larger than 1 cm (p=0.000).

**TABLE 2:** Histopathological features of resected polyps according to size (≤ 1 cm versus ≥1.1 cm)

Histopathology	Polyp size			
	0 to 1 cm		≥1.1 cm	
	N	%	N	%
Tubular adenoma	105	43.6	11	35.6
Villous adenoma	3	1.2	5	16.1
Tubulovillous adenoma	10	4.1	9	29.0
Hyperplastic polyp	59	24.5	4	12.9
Inflammatory polyp	36	15.0	1	3.2
Hamartoma	1	0.4	0	0
Normal mucosa	16	6.6	1	3.2
Other	11	4.6	0	0

Similar findings were observed when the size cutoff was set at 0.5 cm (Tables 3 and 4). Only 8.5% of adenomas ≤0.5 cm in size had a villous component, versus 57.6% of those 0.6 cm or larger (p=0.000).

**TABLE 3:** Characteristics of resected polyps according to size (≤0.5 cm versus ≥0.6 cm)

Polyp characteristics			Polyp size				p
			0 to 0.5 cm		≥0.6 cm		
			N	%	N	%	
Morphology	Sessile		181	91.9	36	48.0	0.000
	Pedunculated		16	8.1	39	52.0	
Site	Distal		120	60.9	48	64.0	0.677
	Proximal		77	39.1	27	36.0	
Histopathology	Adenoma		92	46.7	51	68.0	0.002
	Other		105	53.3	24	32.0	
Dysplasia	Present		91	46.2	49	65.3	0.003
	Grade	Low	86	43.7	36	48.0	
		Moderate	5	2.5	12	16.0	
		High	0	0	1	1.3	
	Absent		103	52.3	23	30.6	
	Indeterminate		3	1.5	3	4.1	

**TABLE 4:** Histopathological features of resected polyps according to size (≤0.5 cm versus ≥0.6 cm)

Histopathology	Polyp size			
	0 to 0.5 cm		≥0.6 cm	
	N	%	N	%
Tubular adenoma	84	42.7	32	42.7
Villous adenoma	3	1.5	5	6.7
Tubulovillous adenoma	5	2.5	14	18.6
Hyperplastic polyp	53	27.0	10	13.3
Inflammatory polyp	27	13.8	10	13.3
Hamartoma	1	0.5	0	0
Normal mucosa	15	7.5	2	2.7
Other	9	4.5	2	2.7

Comparison of polyp histopathology according to patient age showed that subjects aged 50 or older were more likely to have sessile polyps (p=0.023) located in the proximal colon (p=0.009). There were no significant age-related differences in histopathology or presence of dysplasia (Table 5).

**TABLE 5:** Characteristics of resected polyps according to patient age

Polyp characteristics			Patient age				p
			≥50 years		≤49 years		
			N	%	N	%	
Morphology	Sessile		46	69.7	171	83.0	0.023
	Pedunculated		20	30.3	35	17.0	
Site	Distal		50	75.7	118	57.3	0.009
	Proximal		16	24.3	88	42.7	
Histopathology	Adenoma		28	42.4	115	55.8	0.066
	Other		38	57.6	91	44.2	
Dysplasia	Present		27	40.9	113	54.9	0.102
	Grade	Low	21	31.9	101	49.0	
		Moderate	6	9	11	5.4	
		High	0	0	1	0.5	
	Absent		37	56.1	89	43.2	
	Indeterminate		2	3.0	4	1.9	

Histopathological features according to patient age are described in Table 6. There were no statistically significant between-group differences in the presence of a villous component (p=0.511).

**TABLE 6:** Histopathological features of resected polyps according to patient age

Histopathology	Patient age			
	≤49 years		≥50 years	
	N	%	N	%
Tubular adenoma	23	34.8	93	45.1
Villous adenoma	2	3.0	6	2.9
Tubulovillous adenoma	3	4.6	16	7.8
Hyperplastic polyp	23	34.9	40	19.5
Inflammatory polyp	10	15.1	25	12.1
Hamartoma	0	0	1	0.5
Normal mucosa	3	4.6	14	6.8
Other	2	3.0	11	5.3

## DISCUSSION

Colorectal polyps are common, being detected in up to 33% of colonoscopies^9^. Two-thirds of all colon polyps are adenomas, which, by definition, are dysplastic and have the potential for malignant transformation. Nearly all CRCs arise from adenomas, but only a small minority of adenomas will actually progress to cancer^6^.

The incidence of adenomatous polyps has been described as 21-28% in patients aged 50-59 years, 41-45% in the 60-69 age group, and 53-58% in patients over the age of 70^14^. The prevalence of adenomatous polyps on autopsy has been reported as 20-30%, and the incidence of these lesions appears to increase with age^19^. According to current ASGE/ACG recommendations, adenomas will be detected during first-ever colonoscopy in over 25% of asymptomatic men and 15% of asymptomatic women over the age of 50^20^.

The lower incidence of polyps in this study might be explained by the fact that the indication for colonoscopy was not taken into account, and that some of the colonoscopies included were performed under suboptimal bowel preparation conditions. The polyp detection rate depends on a host of variables, including the demographics of the screened population (age, sex, family history of CRS), the quality of bowel prep, endoscopist technique and expertise, and endoscope withdrawal time^9^.

Just over half of all polyps in this series (51%) were solitary. According to Lowenfels et al.^12^, approximately two-third of patients have solitary polyps, and the frequency of larger polyps increases with advancing age.

In this study, 91.9% of polyps <0.5 cm in size were sessile. Conversely, those larger than 1 cm were mostly pedunculated (67.7%). It is well known that polyps<5 mm, also known as minute polyps, are rarely stalked^6^.

Histopathological examination is accepted as the gold standard for definition of polyp size and has been recommended for clinical practice and research purposes alike^5^. In the present study, the polyp size estimated by the endoscopist at the time of resection matched the size later determined by the pathologist in 80.1% of cases. According to Schoen et al.^24^, polyp size is estimated inaccurately by the endoscopist in 20% of cases, with a trend toward overestimation. Conversely, other authors have concluded that endoscopists tend to underestimate lesion size^15^. In this study, polyp size was defined as that estimated by the endoscopist and noted in the colonoscopy report, so that histopathology findings could be interpreted from the point of view of the examining physician, who will be in charge of patient care and follow-up.

The histological features and size of adenomas are the most important determinants of malignant potential^6^. Adenomas may be classified as tubular, villous, or tubulovillous, according to their glandular architecture. Over 80% of colonic adenomas are tubular^16^.

Most polyps resected from the patients in this sample were ≤1 cm in size, left-sided, and had tubular adenoma as the predominant histopathological type, which corroborates previous findings^26^. However, in patients over the age of 50, polyps were most commonly located in the proximal colon. Prior studies have reported age as a major risk factor for proximal lesions^11^. Other authors, however, have found no age-related differences in polyp distribution^17^.

There was a higher incidence of adenoma and dysplasia in patients over the age of 50, but the difference did not reach statistical significance. Other studies have reported a higher incidence of adenomas in general and advanced adenomas in particular after the fifth decade of life^23,18^. There were no significant between-group differences in presence of villous component. Villous polyps may become malignant in 29-70% of cases^13^. The presence of a villous component in endoscopically resected adenomas is a predictor of advanced lesions on follow-up colonoscopy^28^.

Winawer et al.^29^, in an analysis restricted to polyps ≥1 cm in diameter, found that 86% of adenomas exhibited slight atypia, 8% were moderately atypical, and 6% showed marked atypia, also known as carcinoma in situ. In the present study, 54.7% of polyps ≥1 cm were slightly dysplastic, 22.6% were moderately dysplastic, and 3.3% exhibited high-grade dysplasia.

One important finding of this study was the absence of any significant difference in histopathology features when the size cutoff for polyps was set at 0.5 cm or 1.0 cm. In both cases, increasing polyp size was associated with increased odds of adenoma, villous component, and dysplasia. Therefore, one may conclude that small (6-9 mm) polyps should not be neglected.

Few studies have assessed the rate of advanced histology on the basis of polyp size^10^. One such study concluded that removal of a greater number of polyps (including smaller polyps) with a lower rate of adenoma resection is preferable to removal of fewer polyps for a higher rate of adenoma resection^3^.

Kim et al.^7^ reported advanced histology in only 3% of polyps 6-9 mm in diameter. Other studies^30^ found evidence of a villous component in 4-15% and high-grade dysplasia in 4.3-5.8% of polyps in this size range. Lieberman et al.^10^ found a high proportion of advanced histology (prevalence up to 30.6%) in patients with polyps larger than 1 cm, whereas those with small (6-9 mm) polyps were at intermediate risk (6.6%), including of high-grade dysplasia (0.92%).

In another study^27^, which included patients aged 40-89 years, 18.7% of subjects had adenomas, 5% of which were advanced. The prevalence of advanced histology was 85% in polyps ≥1 cm, 27% in polyps 6-9 mm and 10% in polyps ≤5 mm in size. The authors concluded that failure to remove small polyps may place patients at risk of progression to advanced lesions and cancer.

Rex et al.^21^, in a retrospective study of 5079 patients, found advanced histology in 0.87% of minute (≤5 mm) polyps and 5.3% of small (6-9 mm) polyps. Chaput et al.^2^ found advanced histology in 4.7% of minute and 35.2% of small polyps, mostly due to presence of a villous component. The authors noted that polyp size <1 cm was associated with a higher incidence of advanced adenoma.

In a retrospective study of patients undergoing first-ever colonoscopy, Shapiro et al.^25^ found that 1.6% of polyps ≤5 mm exhibited high-great dysplasia or malignant transformation, and 4.1% contained a villous component. The rate of advanced histology for polyps 6-9 mm in size was over 15%. The authors concluded that expectant management of small polyps puts more than 5% of patients at risk of dysplasia progression.

In a systematic review by Hassan et al.^4^, advanced adenomas were identified in 5.6% of minute polyps, 7.9% of small polyps, and 87.5% of large (≥1 cm) polyps. The authors concluded that polypectomy of lesions larger than 6 mm identifies 95% of advanced adenomas. When resection is limited to polyps larger than 10 mm, only 88% of advanced lesions are identified.

## CONCLUSION

Polyp size was associated with the presence of adenomatous and villous components and with dysplasia, whereas patient age was more frequently associated with sessile polyps located proximal to the splenic flexure.
